# Plastic use for food and drinks and related knowledge, attitudes, and practices among a sample of Egyptians

**DOI:** 10.3389/fpubh.2023.1146800

**Published:** 2023-09-28

**Authors:** Fatma Mohamed Hassan, Eman D. El Desouky, Marwa Rashad Salem, Motaze Adel Abdelsabour, Mostafa Alaa Abdelmoneim, Mohamed Mahmoud Elsaieed, Mona Mohamed Ali

**Affiliations:** ^1^Forensic Medicine and Clinical Toxicology, Faculty of Medicine, Cairo University, Cairo, Egypt; ^2^Epidemiology and Biostatistics, National Cancer Institute, Cairo University, Cairo, Egypt; ^3^Public Health and Community Medicine, Faculty of Medicine, Cairo University, Cairo, Egypt; ^4^Kasr Alainy Medical School, Cairo University, Cairo, Egypt; ^5^Forensic Medicine and Clinical Toxicology, Faculty of Medicine, Taif University, Taif, Saudi Arabia

**Keywords:** plastic containers, knowledge, attitude, practice, survey, Egypt

## Abstract

**Introduction:**

Plastic is extensively used in everyday life, particularly for food and beverage containers. The inappropriate use of these containers may lead to the leaching of various chemicals from plastic, such as bisphenol A, phthalate, and styrene, which cause numerous adverse health effects. This study aimed to assess the knowledge, attitudes, and practices toward using plastic for food and drinks among a sample of the Egyptian population.

**Materials and methods:**

A questionnaire was designed based on scientific literature to assess sociodemographic data, knowledge, attitudes, and practices toward the use of plastic for food and drinks. A total of 639 participants were recruited by employing the convenience sampling technique.

**Results:**

More than half of the participants (347, 54%) had poor knowledge scores. Personal experiences, social media, and web pages represented the most common knowledge sources. A comparison between plastic-related knowledge scores and the studied sociodemographic characteristics revealed statistically significant differences in age, gender, education, marital status, residence, working, and socioeconomic standard. A good attitude was reported by the majority (515, 80.6%) of participants. The majority (493, 77.2%) were occasional and frequent plastic users and the practice scores were significantly associated with age, education, residence, and socioeconomic standard. Higher educational level, gender (women), and rural residence were predictors of good participants knowledge, while lower socioeconomic status and urban residence were predictors of bad participants practice in a multivariate logistic regression analysis.

**Conclusion:**

The observed unsatisfactory knowledge and practice scores vs. the high attitude indicates a knowledge gap that can help direct future improvements. We call for public awareness programs about safe plastic use and the related health hazards of plastic chemicals. We also stress upon the urgent need for a collaboration between health authorities and the plastic and food industry to guarantee that information about proper plastic use is conveyed to consumers.

## Introduction

Plastic has become an integral part of human life because it is a low-cost, lightweight, and long-lasting material that can be molded into a wide variety of products. Food and beverage containers, as well as food packaging materials, are examples of these products (1). Egypt consumes 0.7% of the world's plastic production and 11% of the Middle East's share. Furthermore, the plastic market in Egypt is expected to expand at a rate of 10% a year over the next 10 years, making Egypt the top plastic consumer in Africa (2).

Plastics are made of a wide range of chemicals; plastic packaging alone has been linked to more than 4,000 chemicals. Phthalates and bisphenol A (BPA) are among the chemicals used in plastic that are susceptible to leaching (3). Numerous studies have reported an association between phthalates and BPA with several adverse health conditions, such as polycystic ovarian disease (4, 5), frequent abortion (6), obesity (7), and cancer (8, 9).

A recent study investigated the habits related to food packaging in a sample of Portuguese citizens and their knowledge and concerns about its use, with the majority confirming that they think about the negative impact of plastic packaging. Most of the interviewees had concerns about the use of plastic packaging, and 55% reported that they are attempting to change their habits to avoid the use of plastics in this context (10). In addition, several studies in different countries reported poor knowledge and practices of individuals regarding the use of plastic for food and drinks (3, 11, 12). In that context, the United Nations Environment Programme statement suggested a 10-step plan for decision-makers. Increasing user awareness is one of them (13). This study aimed to assess the knowledge, attitudes, and practices toward the safe use of plastic containers for food and drinks.

## Materials and methods

### Study design

The proposed study was an exploratory cross-sectional study that was performed among a sample of the general population in Egypt to assess the knowledge, attitudes, and practices toward the use of plastic containers for food and drinks. The research was carried out following the guidelines outlined in the Checklist for Reporting Results of Internet E-surveys for the online survey (14) and the Strengthening the Reporting of Observational Studies in Epidemiology (STROBE) Statement for reporting observational studies (15).

### Sample size and sampling technique

The researchers used a consecutive convenience sampling technique called “self-selection web-based questionnaires” and shared the questionnaire link with groups on Facebook, the most frequently utilized social media in Egypt. Requests were sent to the administrators of these groups to obtain permission to distribute this survey. The researchers posted the survey link along with an encouraging statement about its purpose and the contact information of one of the researchers. The questionnaire was open to the public, so anyone could participate. The researchers also printed and distributed the questionnaire to study participants who could not access the Google Forms. The total returned complete questionnaires were 204 out of 250 printed copies and 435 online forms collected via a Google Forms link sent through social media. The final total was 639 questionnaires. The inclusion criteria for participants were as follows: (i) being an Egyptian resident; adults (18 years old); and willing to participate between 1st of October 2022, and 1st of December 2022. Open Epi was used to calculate the required sample size, using the following formula: (*n* = [DEFF^*^Np (1-p)]/[(d2/Z21-/2^*^(N-1) + p^*^(1-p)]).

In the formula, n refers to the required sample size, *Z*_∝/2_ = 2.57(99% CI), P refers to the prevalence of the outcome (good knowledge assumed to be 50%), N refers to population size (for the finite population correction factor or FPC), d refers to the margin of error; 0.05, and DEFF refers to the design effect (for cluster surveys, here assumed to be 1). With a precision of 5%, a 95% confidence interval, and an 80% power, the minimal sample size required is 384 participants. Adding 50% to compensate for potential non-responses, the minimal sample size was estimated to be 576 participants.

### Data collection technique

We used the online data collection method. A form was created via Google Forms, and participants were invited to complete and submit it. The researchers also printed and distributed the questionnaire to study participants who could not access the Google Forms. The questionnaire consisted of the following sections:

- Sociodemographic characteristics: Age, gender, occupation, education, residence, marital status, and socioeconomic level.
- The knowledge of study participants about plastic use consisted of a total of 11 items. The questions were formatted as close-ended statements with yes, no, and do not know options.
- The attitudes of study participants, which were assessed using five closed-ended questions with options of strongly agree, agree, neutral, strongly disagree, and disagree.
- The practice part, which had 7 items, assessed the frequency of plastic use-related practice in its weekly format and the selection of food and drink containers.
- Sources of knowledge, which were assessed through multiple options, including social media, web pages, newspapers, study materials, and parents.

Questions used in these sections were adopted from available studies in the literature (3, 16).

### Pilot test

The data collection instrument for the survey was tested on a subset (58, 10%) of the total sample size, to evaluate the questionnaire's suitability in terms of language, questions, and time needed to complete it, as well as to investigate any variations between online and offline responses collected by the interviewers. The required modifications were applied; for example, some questions such as “What are the most common types of plastic products that you use?” were deleted. The questionnaire content was validated by four faculty members who are experts in public health, and the required modifications were made.

A Cronbach's alpha reliability test was performed for different sections of the questionnaire, as well as for the entire questionnaire. The results were as follows: knowledge section = 0.84, attitude section = 0.77, practice section = 0.65, and entire questionnaire = 0.72.

### Statistical analysis

Data entry was carried out using the Statistical Package for Social Science (SPSS) version 28.0 (IBM, SPSS, USA). Categorical variables were expressed in numbers and percentages. Comparisons were made by performing a chi-squared test. Quantitative variables were examined for normality by performing the Kolmogorov–Smirnov test and were expressed using mean and standard deviation; a *t*-test and one-way ANOVA followed by the Bonferroni *post-hoc* test were performed for comparison, as appropriate. A logistic regression analysis was performed to assess the effects of different factors on the likelihood of participants having good knowledge or exhibiting poor practice. All tests were two-tailed, and a *P-*value of <0.05 was considered statistically significant.

Regarding knowledge about plastic containers for food and drink, the points for questions were distributed as follows: No and I don't know = 0 and Yes = 1; the total score was 11, and the range of scores was 0–11. Participants with scores of 9–11 (>75%) were considered “good,” those with scores of 6–8 (>50%) were considered “fair,” and those with scores of 1–5 (50%) were considered “poor.” For the attitude section, the highest score was 5 for strongly agree and 1 for strongly disagree. The total attitude score was 25, and the range of scores was 5–25. Participants with scores of 19–25 (>75%) were considered “good,” those with scores of 13–18 (>50%) were considered “fair,” and those with scores of 5–12 (50%) were considered “poor.”

Regarding practices related to plastic containers for food and drink, the points for questions were distributed as follows: Not at all = 0 and Usually = 3, except for reversed questions, where the points were Not at all = 3 and Usually = 0. The total score was 21, and the range of scores was 0–21. Participants with a score of 16–21 (>75%) were classified as “frequent users” of plastic food and beverage products, those with a score of 11–15 (>50%) were classified as “occasional users,” and those with a score of 0–10 (50%) were classified as “rare users.” A higher score indicated bad practice, while a lower score indicated good practice (16).

### Ethical considerations

The National Cancer Institute Ethical Review Board at Cairo University granted ethical approval for the study protocol (Approval number = 2207-504-012). All procedures for data collection were treated with confidentiality according to the Helsinki Declaration on biomedical ethics. Participants were informed that the survey was anonymous and that participation was voluntary. Only those who agreed were included in the study. All procedures for data collection were treated according to the Helsinki Declaration and Biomedical Ethics (17). All the study participants provided written informed consent; for the online form, they provided electronically signed informed consent.

## Results

The demographic characteristics of the study participants are demonstrated in Table 1. Six hundred thirty-nine individuals completed the questionnaire; their mean age was 31.0 ± 12.8 years (range 18–73 years), with 356 (55.8%) being <31 years old and 327 (51.2%) being women. The majority of participants (495, 77.4%) were highly educated (university and postgraduate levels), approximately half of them (324, 50.7%) were single, 497 (77.8%) were from urban areas, and more than three-quarters (501, 78.4%) reported a medium socioeconomic standard. More than half were working (324, 50.7%).

**Table 1:** Demographic characteristics of the study participants (*n* = 639).

**Characteristics**		***n* (%)**
Age (yrs.)	18–20	160 (25.1)
	21–30	196 (30.7)
	31–40	121 (18.9)
	41–50	103 (16.1)
	≥51	59 (9.2)
Gender	Women	327 (51.2)
	Men	312 (48.8)
Education	Basic education (primary-preparatory)	40 (6.3)
	Secondary (general-technical)	104 (16.3)
	University	365 (57.1)
	Postgraduate	130 (20.3)
Marital status	Single	324 (50.7)
	Married	288 (45.1)
	Divorced	19 (3.0)
	Widow	8 (1.3)
Residence	Urban area	497 (77.8)
	Rural area	142 (22.2)
Socioeconomic status^*^	Low	39 (6.1)
	Medium	501 (78.4)
	High	99 (15.5)
Working	No	315 (49.3)
	Yes	324 (50.7)

* Self-reported socioeconomic status.

### Knowledge of participants

The items used for knowledge assessment and the frequency of participants' responses are presented in Table 2. We found that only 235 (36.8%) participants have correct knowledge about the safe use of plastic containers for eating or storing cold food or drink. However, most of the participants (539, 84.4%) have correct knowledge about handling hot food and drinks in plastic containers. Regarding the numbers written on plastic containers, 207 (32.4%) participants reported that they knew their meanings, but only approximately 16% of the participants correctly answered the questions related to these numbers. As for overall knowledge, more than half (347, 54.3 %) of them had poor knowledge scores. Their mean knowledge score was 5 ± 2 (range 0–11).

**Table 2:** Distribution of knowledge assessment items regarding the use of plastic containers for food and drinks among study participants (*n* = 639).

	**Knowledge items**	**Wrong answer/lack of knowledge *n* (%)**	**Correct answer *n* (%)**
1	Plastic containers can be used to safely eat or store cold foods or drinks	404 (63.2)	235 (36.8)
2	Plastic containers can be used to safely eat or store hot foods or drinks	100 (15.6)	539 (84.4)
3	Cooking hot food in plastic materials is dangerous and harmful to health.	59 (9.2)	580 (90.8)
4	Do you think that eating hot food in plastic containers could be related to an increased risk of cancer?	228 (35.7)	411 (64.3)
5	White or clear plastics are of better quality and safer than colored plastics.	329 (51.5)	310 (48.5)
6	Plastic can be used safely for heating food in the microwave.	205 (32.1)	434 (67.9)
7	Plastic wraps used for food packaging contain chemicals harmful to human health.	353 (55.2)	286 (44.8)
8	The plastic wrap used to preserve food should not come in direct contact with it but should be placed at least 1 in away from the food.	372 (58.2)	267 (41.8)
9	Regarding the numbers and symbols on plastic containers, do you know their meaning?	432 (67.6)	207 (32.4)
10	Regarding the numbers and symbols on plastic containers, the larger the number written on the bottom of the plastic, the less likely it is to harm the health.	533 (83.4)	106 (16.6)
11	Regarding the numbers and symbols on plastic containers, marks 1 to 5 are suitable for food preservation.	532 (83.3)	107 (16.7)
	Total knowledge score		
	Good	49 (7.7)	-
	Fair	243 (38.0)	-
	Poor	347 (54.3)	-
	Mean ± SD	5.0 ± 2.0	-
	Range	0–11	-

SD, standard deviation.

**Figure 1:**
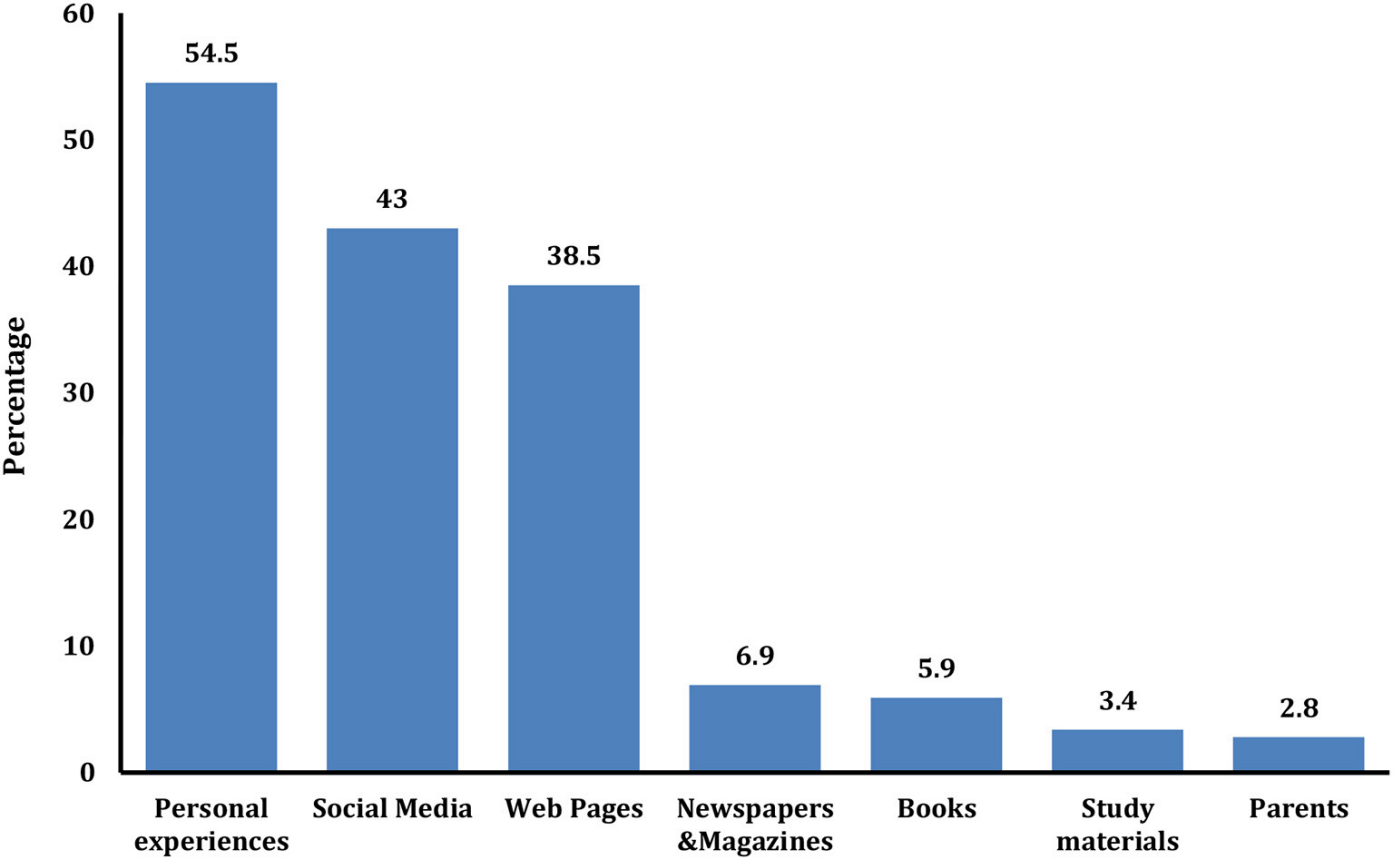
Sources of plastic-related knowledge among the enrolled participants (*n* = 639).

Figure 1 shows the knowledge sources of the participants, where personal experiences, social media, and web pages represented the most common sources (54.5, 43.0, and 38.5%, respectively). Parents were the least-reported source.

### Attitude of participants

More than three-fourths of the participants showed a positive attitude toward the use of plastic containers for attitude statements regarding food and drinks. Most of them were against using plastic with hot drinks or foods, which should be avoided, and were willing to pay more money to buy alternative materials to such as plastic (Table 3). Overall, a good attitude was reported by the majority (515, 80.6%) of participants. The participants' mean attitude score was 21 ± 3 (range 11–25).

**Table 3:** Distribution of attitude assessment items regarding the use of plastic containers for food and drinks among study participants (*n* = 639).

	**Attitude items**	**Strongly disagree *n* (%)**	**Disagree *n* (%)**	**I don't know *n* (%)**	**Agree *n* (%)**	**Strongly agree *n* (%)**
1	Chemicals in plastic containers can transfer to the foods and drinks you eat.	2 (0.3)	23 (3.6)	76 (11.9)	232 (36.3)	306 (47.9)
2	The use of plastic with hot drinks or foods should be avoided.	4 (0.6)	40 (6.3)	37 (5.8)	268 (41.9)	290 (45.4)
3	The consumption of plastic containers should be reduced.	0	13 (2.0)	111 (17.4)	221 (34.6)	294 (46.0)
4	I am willing to pay more money to buy alternative materials to plastic.	3 (0.5)	44 (6.9)	149 (23.3)	234 (36.6)	209 (32.7)
5	More information should be published about the health effects of using plastic containers.	1 (0.2)	9 (1.4)	105 (16.4)	177 (27.7)	347 (54.3)
	Overall attitude score					
	Good	515 (80.6)				
	Fair	122 (19.1)				
	Poor	2 (0.3)				
	Mean ± SD	21.0 ± 3.0				

SD, standard deviation.

### Practices of participants

**Figure 2:**
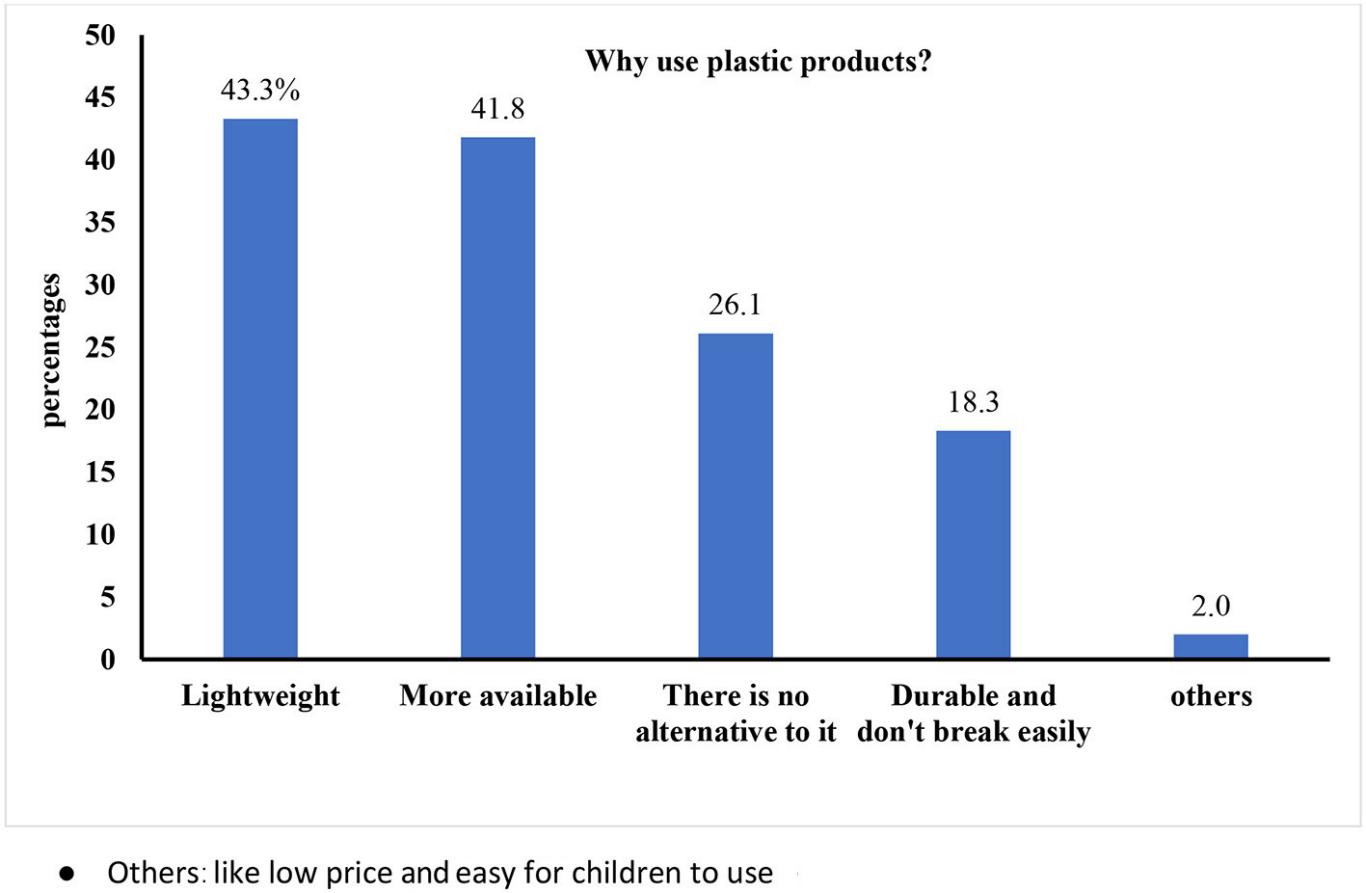
The most stated reason for using plastic among the enrolled participants (*n* = 639).

As shown in Figure 2, the most stated reason for using plastic was its light weight (43.3%), followed by its availability (41.6%).

As displayed in Table 4, more than half (320, 50.1%) of the enrolled participants reported that they usually eat or drink in plastic containers, and 358 (56.0%) participants reported that they reuse plastic bottles to save or drink water. More than half (357, 55.9%) of the participants do not use plastic to heat food in the microwave, and nearly half (271, 42.4%) of the participants do not check the type of plastic before purchasing. Most (493, 77.2%) of the participants were occasional and frequent users. The mean practice score was 11.7 ± 3.5 (range 1–21).

**Table 4:** Distribution of practice assessment items regarding the use of plastic containers for food and drinks among study participants (*n* = 639).

	**Practice items**	**Not at all *n* (%)**	**Occasionally (1–2 days/week) *n* (%)**	**Sometimes (3–4 days/week) *n* (%)**	**Usually (5–7 days/week) *n* (%)**
1	How often do you eat or drink in plastic containers?	14 (2.2)	211 (33.0)	94 (14.7)	320 (50.1)
2	How often do you use plastic containers to eat or keep hot foods or drinks?	150 (23.5)	182 (28.5)	229 (35.8)	78 (12.2)
3	How often do you reuse plastic bottles to save or drink water?	46 (7.2)	151 (23.6)	84 (13.1)	358 (56.0)
4	How often do you put plastic wrap on food containers to store leftovers?	61 (9.5)	143 (22.4)	181 (28.3)	254 (39.7)
5	How often do you drink water from plastic bottles kept in a hot place, such as a car, for a long time?	349 (54.6)	142 (22.2)	45 (7.0)	103 (16.1)
6	How often do you use plastic to heat food in the microwave?	357 (55.9)	82 (12.8)	48 (7.5)	152 (23.8)
7	How often do you check the type of plastic before buying?^*^	271 (42.4)	279 (43.7)	9 (1.4)	80 (12.5)
	Overall practice score				
	Rare users	146 (22.8)			
	Occasional users	384 (60.1)			
	Frequent users	109 (17.1)			
	Mean ± SD	11.7 ± 3.5			

* Reversed question; SD, standard deviation.

A comparison between plastic-related knowledge scores and the studied sociodemographic characteristics revealed statistically significant differences in age, gender, education, marital status, residence, working, and socioeconomic standard, as displayed in Table 5. Regarding practices, the same significant differences were revealed, except for gender, marital, and working status, which were not significant.

**Table 5:** Relationship between the distribution of knowledge and practice scores regarding the safe uses of plastic containers and demographic characteristics.

**Demographic characteristics**		**Knowledge grade**			***p*-value**	**Practice grade**		***p*-value**
		**Poor users** ***n** =* **347**		**Fair-Good users** ***n** =* **292**		**Rare users** ***n** =* **146**	**Occasional-Frequent users**, ***n** =* **493**	
		***n***	***n*** **(%)**	***n*** **(%)**		***n*** **(%)**	***n*** **(%)**	
Age groups	18–20	160	69 (43.1)	91 (56.9)	< 0.001	39 (24.4)	121 (75.6)	0.005
	21–30	196	97 (49.5)	99 (50.5)		55 (28.1)	141 (71.9)	
	31–40	121	64 (52.9)	57 (47.1)		32 (26.4)	89 (73.6)	
	41–50	103	74 (71.8)	29 (28.2)		15 (14.6)	88 (85.4)	
	≥51	59	43 (72.9)	16 (27.1)		5 (8.5)	54 (91.5)	
Gender	Women	327	158 (48.3)	169 (51.7)	0.002	79 (24.2)	248 (75.8)	0.419
	Men	312	189 (60.6)	123 (39.4)		67 (21.5)	245 (78.5)	
Education	Basic -secondary	144	135 (93.8)	9 (6.3)	< 0.001	0 (0.0)	144 (100.0)	< 0.001
	University-postgraduate	495	212 (42.8)	283 (57.2)		146 (29.5)	349 (70.5)	
Marital status	Divorced	19	15 (78.9)	4 (21.1)	0.001	4 (21.1)	15 (78.9)	0.078
	Married	288	173 (60.1)	115 (39.9)		56 (19.4)	232 (80.6)	
	Single	324	153 (47.2)	171 (52.8)		86 (26.5)	238 (73.5)	
	Widow	8	6 (75.0)	2 (25.0)		0 (0.0)	8 (100.0)	
Working	No	315	151 (47.9)	164 (52.1)	0.001	74 (23.5)	241 (76.5)	0.702
	Yes	324	196 (60.5)	128 (39.5)		72 (22.2)	252 (77.8)	
Residence	Rural area	142	46 (32.4)	96 (67.6)	< 0.001	48 (33.8)	94 (66.2)	< 0.001
	Urban area	497	301 (60.6)	196 (39.4)		98 (19.7)	399 (80.3)	
Socioeconomic status	High	99	42 (42.4)	57 (57.6)	0.010	37 (37.4)	62 (62.6)	< 0.001
	Low-medium	540	305 (56.5)	235 (43.5)		109 (20.2)	431 (79.8)	

*P*-value < 0.05 is considered significant.

Age group (18–20 vs. 41–50), gender (women), higher education (university–postgraduate), residence in a rural area, and a high socioeconomic level were significantly associated with a higher mean knowledge score regarding the safe uses of plastic containers (*p* < 0.05), as illustrated in Table 6. Concerning the safe use of plastic, women and participants with basic and secondary education, those living in cities, and those working were significantly associated with a higher mean attitude score (*p* < 0.001). Contrarily, being single (vs. others) and age (21–30 vs. 31–40 and 41–50) were associated with a lower attitude score. Statistically significant relationships were revealed between the mean practice scores and all the studied demographic characteristics (age, education, marital status, and working), except for gender and residence. Age (18–20 vs. 21–30 and 31–40) had a higher practice score; the 31–40 age group had a lower practice score than those aged ≥51 years. Widowed participants had a higher score than divorced participants.

**Table 6:** Relationship between the mean scores of knowledge, attitude, and practice regarding the safe uses of plastic containers and demographic characteristics (*n* = 639).

		**Knowledge**		**Attitude**		**Practice**	
		**Mean** ±**SD**	***P*** **-value**	**Mean** ±**SD**	***P*** **-value**	**Mean** ±**SD**	***P*** **-value**
Age groups	18–20^a^	6 ± 2^d^	0.013	19 ± 2.4	< 0.001	12.5 ± 3.5^b^^,^ ^c^	< 0.001
	21–30^b^	5 ± 2		21 ± 2.7^c^^,^ ^d^		11.35 ± 3.8	
	31–40^c^	6 ± 2		22 ± 2.3		10.67 ± 3.2^e^	
	41–50^d^	5 ± 2		22 ± 2.2		11.72 ± 3.4	
	≥51^e^	5 ± 2		22 ± 2.7		12.29 ± 3.1	
Gender	Women	6 ± 2	< 0.001	22 ± 2.6	< 0.001	11.4 ± 3.5	0.063
	Men	5 ± 2		20 ± 2.7		11.92 ± 3.6	
Education	Basic-secondary	4 ± 1	< 0.001	23 ± 2	< 0.001	13.33 ± 2.1	< 0.001
	University-postgraduate	6 ± 2		21 ± 3		11.17 ± 3.7	
Marital Status	Divorced^f^	5 ± 2	0.317	22 ± 2	< 0.001	10.84 ± 2.9^i^	0.040
	Married^g^	5 ± 2		22 ± 2.4		11.35 ± 3.5	
	Single^h^	5 ± 2		20 ± 2.5^f^^,^ ^g^^,^ ^i^		11.91 ± 3.7	
	Widow^i^	6 ± 2		23 ± 2.1		14 ± 2.1	
Residence	Rural area	6 ± 2	< 0.001	20 ± 2.7	< 0.001	11.42 ± 4	0.360
	Urban area	5 ± 2		21 ± 2.6		11.72 ± 3.4	
Socioeconomic standard	High	6 ± 2	0.025	21 ± 3	0.514	10.67 ± 4.3	0.002
	Low-medium	5 ± 2		21 ± 3		11.84 ± 3.4	
Working	No	6 ± 2	0.506	20 ± 2.7	< 0.001	12.22 ± 3.5	< 0.001
	Yes	5 ± 2		22 ± 2.5		11.11 ± 3.5	

Each age and marital status category has been assigned a letter:

a 18–20 years,

b 21–30 years,

c 31–40 years,

d 41–50 years,

e ≥51 years,

f divorced,

g married,

h single, and

i widow. Letters present above the group mean indicate significance with the assigned groups. A *p*-value of < 0.05 is considered significant.

A higher education level, gender (women), and residence in rural areas were found to be predictors of good participant knowledge in a multivariate logistic regression analysis. Participants with a higher level of education were 22.2 times more knowledgeable than those with a primary or secondary education. Women outperformed men in terms of knowledge, with an OR of 3.0 and a 95% confidence interval (2.1–4.3). Participants living in rural areas had 2.6 times more knowledge than those living in urban areas (Table 7).

**Table 7:** Multivariate logistic regression analysis for factors associated with good knowledge and bad practice regarding the use of plastic containers for food and drinks.

					**95% C.I. for OR**	
**To detect (fair and good knowledge)**	**B**	**S.E**.	***p*** **-value**	**OR**	**Lower**	**Upper**
Education (university and postgraduate/basic and secondary)	3.10	0.37	< 0.001	22.2	10.8	45.6
Gender (women/men)	1.09	0.19	< 0.001	3.0	2.1	4.3
Residence (rural/urban)	0.96	0.22	< 0.001	2.6	1.7	4.1
Constant	−3.56	0.38	< 0.001	0.0		
**To detect (occasional and frequent users) bad practice**						
SES (low and medium/high)	0.97	0.24	< 0.001	2.6	1.6	4.2
Residence (urban/rural)	0.83	0.22	< 0.001	2.3	1.5	3.5
Constant	−0.19	0.28	0.490	0.8		

B, Regression coefficients; SE, Standard error of the coefficient; OR, Odds Ratio, and 95% CI for OR = 95% confidence interval for the Odds Ratio. SES, Socioeconomic status. *P*-value < 0.05 is considered significant.

Participants living in urban areas followed bad practices regarding the use of plastic containers for food and drinks 2.3 times more times than those living in rural areas. Participants with a low to medium socioeconomic standard followed bad practices 2.6 times more times than those with a higher standard.

## Discussion

This study revealed that the majority of the participants had unsatisfactory knowledge and improper practices regarding the appropriate use of plastic for foods and drinks, which necessitates the implementation of public awareness programs about the safe uses of plastic. This finding is frustrating because knowledgeable people are more concerned about environmental pollution and engage in protective behaviors. Coco Chin et al. (18) found the same results among Malaysians.

Similarly, El-sayed et al. (1) reported an unsatisfactory knowledge level among 120 Egyptian children's mothers. Kasemsup and Neesanan (16), in their study that included 100 parents, found that more than 80% of participants lacked adequate knowledge about appropriate plastic use. Furthermore, Kaur et al. (19) assessed the knowledge about health risks associated with plastic use among students and observed that, at the pre-test, 60% of them had poor knowledge, while none had good knowledge. In addition, Samuel (20) studied the knowledge level of street food sellers and customers in Nigeria and found that 89.3% of the food sellers and 59.3% of the customers do not know the health hazards of wrapping hot food in plastic or cooking food using plastic. Recently, a community-based study conducted in Egypt by Hamza and Mahmoud (21) revealed that only 24% of the participants had adequate knowledge regarding single-use plastics.

Contrarily, Vigneshwaran and Arun Kumar (22) reported that three-fourths of their study participants had a high knowledge level regarding plastic use in Tiruchirappalli Municipal Corporation, Tamil Nadu, India. Additionally, Praveena found that 70% of the participants had moderately adequate knowledge of Mohan Kumar Nagar, Bengaluru, India (11).

This may be justified by the fact that India is a country that produces a tremendous number of plastic products, so Indian people have a high basic knowledge level.

One of the knowledge assessment items was symbols on plastic containers; only approximately 16% of our study participants knew their correct meanings. According to Nourbakhsh et al. (3), an average of 4.83% of their study participants in Iran verified their exact knowledge of various plastic labels. Unawareness of labels used at the bottom of plastic containers can be a barrier against proper use. According to the Food and Drug Administration (FDA), symbols number 1, 2, 4, and 5 (PET, HDPE, LDPE, and PP, respectively) are considered safe food-contact plastics (23).

Regarding attitude scores, our results revealed good attitude levels among the participants. More than 80% of the participants approved that the consumption of plastic containers should be reduced and that information about the health hazards of using plastic containers for food and drinks should be disseminated. It must be noted that attitude scores were higher than knowledge scores. Similarly, Kasemsup and Neesanan (16) observed this discrepancy between knowledge and attitude regarding plastic use, and they attributed this discrepancy to the fact that consumers are aware of the harmful effects of using plastic but need more information about the appropriate use of plastic containers for food and drinks, including different types of plastics, symbols and their specification, and microwave use. This finding is parallel to the outcomes of multiple studies (21, 24).

Regarding practice assessment items, approximately 76.5% of our study participants used plastic containers for hot foods or drinks with variable frequency. Similarly, Alharbi et al. (25), in their study that involved Saudi pregnant women, observed that almost 70% of the participants reheat or buy hot food in plastic containers. Additionally, we found that more than 90% of our study participants reused plastic bottles for saving and drinking water. This result is higher than that obtained in the study by El-sayed et al. (1), where 80.8% of pre-test mothers used natural water bottles more than once. Furthermore, Kasemsup and Neesanan (16) reported that 74.5% of their participants reused plastic bottles.

Plastic water bottles are usually made of polyethylene terephthalate (PET). The repeated use of such water bottles has a risk of bacterial or fungal growth inside the bottle and migration of chemicals from the inner surface of the bottle into water. Several compounds, including acetaldehyde, antimony, and phthalates, are suspected to leach from PET and lead to adverse health effects among consumers (26, 27).

Our results revealed that most participants (91.5%) use plastic wrap in variable frequencies to keep leftovers. Similarly, in a study involving 1,000 European citizens that evaluated awareness of the direct and indirect effects of plastics on human health, food packaging was the most commonly (*n* = 920, 92.5%) used modality of plastics (28). Additionally, Du Preez et al. (29) reported a high frequency of using plastic food packaging among South African young adults; 58.1% of the participants used it daily, and 23.0% used it more than once a week.

Regarding practice level, the majority of participants (77.2%) were occasional and frequent plastic users. Similarly, El-sayed et al. (1) found that 95.8% of pre-test Egyptian participants had improper practices regarding safe plastic use. Furthermore, Kasemsup and Neesanan (16) reported that most of their respondents usually use plastic for food and drinks.

In contrast to our findings, Vigneshwaran and Arun Kumar (22) reported high practice levels among participants, which could be attributed to the high basic knowledge of Indian people about plastic use.

Studying the relationship between participants' scores and their sociodemographic characteristics revealed that age and educational level significantly affected all scores (as the educational level increased, the scores increased). This is in agreement with El-Sayed et al. (1), who found that highly educated children's mothers had high mean knowledge, attitude, and practice scores regarding the safe use of plastic containers. We found that participants of younger ages had higher knowledge scores. This could be related to the fact that these ages are the most common users of the Internet and social media, as the participants reported these as the most frequent sources of information. For Facebook (the main social media platform in Egypt), 58.3% of users are in the 18–34 age group (30). This finding highlighted the importance of using Facebook to disseminate health education about the safe uses of plastic and the dangers of improper use and recycling. Furthermore, as reported by Filho et al. (31), factors such as educational background and age play a significant role in determining the level of engagement in reducing plastic usage and the actions undertaken. Any interventions aimed at reducing single-use plastics need to consider these factors.

## Conclusion and recommendations

Based on the study findings, we conclude that the majority of the participants lacked satisfactory knowledge and followed improper practices regarding the safe use of plastic for food and drinks. Therefore, public awareness of appropriate plastic use for food and drinks should be raised. Initiatives for raising public awareness should be supported by various approaches that ensure easy accessibility of information. An efficient low-cost information approach could include the exhibition of banners about the safe uses of plastic for food and drinks and posting pamphlets that display numbers and symbols written on plastic containers near checkouts or cash registers in supermarkets and other retail establishments. Furthermore, television and radio can support broad information dissemination. In addition, continuous health education programs regarding the safe use of plastics should be provided by healthcare providers such as nurses, village health advisors, and midwives. Additionally, we recommend that health authorities, in collaboration with the food industry, evaluate, and regulate the use of plastics. Eventually, policies regulating materials used in plastic manufacture need to be reconsidered to safeguard consumers.

### Study limitations

The current study findings should be viewed in light of the following limitations: The non-probability sampling technique was applied in the current research due to the difficulty of using probability sampling, especially because part of the research part was conducted online. Moreover, a small percentage of the study participants were older than 50 years, and most of them were young due to the frequent use of the Internet and social media among the young generation. However, the researchers conducted the current research to explore the situation in this new area of inquiry and to generate hypotheses, as no information is available about the current research question in Egypt. The intent was not to generalize the study's findings.

## Data availability statement

The raw data supporting the conclusions of this article will be made available by the authors, without undue reservation.

## Ethics statement

The studies involving human participants were reviewed and approved by National Cancer Institute. The patients/participants provided their written informed consent to participate in this study.

## Author contributions

FH conceived the study. MMA contributed to the literature searches and data management. EE and MS contributed to the data analysis and writing of the results. MAbdels, MAbdelm, and ME contributed to the data collection and writing. All authors participated in data collection, drafting, and approving the final manuscript, and they contributed to the article and approved the submitted version.

## References

[1] El-sayed Y Marzouk SA Mahmoud TM El Magrabi NM. Effectiveness of educational intervention on knowledge, attitude and practices of children's mothers regarding the safe use of plastic containers. Am J Nurs Res. (2019) 7:723–731. 10.12691/ajnr-7-5-6

[2] UNIDO. Study on plastic value chain in Egypt. (2021). Available online at: https://www.unido.org/sites/default/files/files/2022-01/Plastic_value_chain_in_Egypt.pdf (accessed December 2022).

[3] Nourbakhsh S Afzal-Aghaee M Salmani ER Naderi M Zangi R Feizi R. Knowledge and behavior assessment about the use of disposable plastic containers amongst medical sciences students in northeastern Iran in 2016. Iranian J Health, Safety Environ. (2017) 4:804–11.

[4] Rutkowska A Rachoń D. Bisphenol A (BPA) and its potential role in the pathogenesis of the polycystic ovary syndrome (PCOS). Gynecol Endocrinol. (2014) 30:260–5. 10.3109/09513590.2013.871517 24397396

[5] Akin L Kendirci M Narin F Kurtoglu S Hatipoglu N Elmali F. Endocrine disruptors and polycystic ovary syndrome: phthalates. J Clin Res Pediatr Endocrinol. (2020) 12:393–400. 10.4274/jcrpe.galenos.2020.2020.0037 32431137 PMC7711640

[6] Liao KW Kuo PL Huang HB Chang JW Chiang HC Huang PC. Increased risk of phthalates exposure for recurrent pregnancy loss in reproductive-aged women. Environ Pollut. (2018) 241:969–77. 10.1016/j.envpol.2018.06.022 30029331

[7] Kim KY Lee E Kim Y. The association between bisphenol a exposure and obesity in children-a systematic review with meta-analysis. Int J Environ Res Public Health. (2019) 16:2521. 10.3390/ijerph16142521 31311074 PMC6678763

[8] Gao H Yang BJ Li N Feng LM Shi XY Zhao WH Liu SJ. Bisphenol A and hormone-associated cancers: current progress and perspectives. Medicine (Baltimore). (2015) 94:e211. 10.1097/MD.0000000000000211 25569640 PMC4602822

[9] Chuang SC Chen HC Sun CW Chen YA Wang YH Chiang CJ. Phthalate exposure and prostate cancer in a population-based nested case-control study. Environ Res. (2020) 181:108902. 10.1016/j.envres.2019.108902 31785779

[10] Weber Macena M Carvalho R Cruz-Lopes LP Guiné RPF. Plastic food packaging: perceptions and attitudes of Portuguese consumers about environmental impact and recycling. Sustainability. (2021) 13:9953. 10.3390/su13179953

[11] Praveena BG. A study to assess the knowledge regarding health hazards of plastic in domestic use and attitude toward the use of alternatives in women residing at Mohan Kumar Nagar, Bengaluru. Int J Nur Med Invest. (2019) 4:50–3. 10.31690/ijnmi/50

[12] Srinivasan N Swarnapriya V. Assessment of knowledge and practice on plastics among the professional course students of Annamalai University, Tamil Nadu. Int J Commun Med Public Health. (2019) 6:510–4. 10.18203/2394-6040.ijcmph20190099

[13] UNEP. Single-use plastics: A Roadmap for Sustainability (2018). (accessed December 2022).

[14] Eysenbach G. Improving the quality of web surveys: the checklist for reporting results of internet E-surveys (CHERRIES). J Med Internet Res. (2004) 6:e34. 10.2196/jmir.6.3.e34 15471760 PMC1550605

[15] Cuschieri S. The STROBE guidelines. Saudi J Anaesth. (2019) 13:S31–4. 10.4103/sja.SJA_543_18 30930717 PMC6398292

[16] Kasemsup R Neesanan N. Knowledge, attitudes and practices relating to plastic containers for food and drinks. J Med Assoc Thai. (2011) 94 Suppl 3:S121–5. 22043764

[17] Carlson VR Boyd KM Webb DJ. The revision of the Declaration of Helsinki: past, present, and future. Br J Clin Pharmacal. (2004) 57:695–713. 10.1111/j.1365-2125.2004.02103.x 15151515 PMC1884510

[18] Coco Chin KK Mahanta J Nath TK. Knowledge, attitude, and practices toward plastic pollution among malaysians: implications for minimizing plastic use and pollution. Sustainability. (2023) 15:1164. 10.3390/su15021164

[19] Kaur S Jeganathan J Kaur M. Effectiveness of structured teaching programme on knowledge regarding health hazards of plastic use among students -a Quasi experimental study. Nurs Health Care Int J. (2019) 3:000180. 10.23880/NHIJ-16000180

[20] Samuel OO. Health hazard of plastic: assessing the knowledge level among street food sellers and customers in Delta State, Nigeria. J Biol Agric Healthcare. (2018) 8:7–13.

[21] Hamza WS Mahmoud SR. A community-based cross-sectional study exploring knowledge, attitude, and practice of adults towards the use and hazards of plastic products. Egyptian J Commun Med. (2023) 41:101–10. 10.21608/ejcm.2022.157402.1232

[22] Vigneshwaran R Arun kumar B. Knowledge attitude and practice on plastic usage among the residents of Tiruchirappalli municipal corporation, Tamil Nadu -A descriptive study IOSR. J Human Soc Sci. (2014) 12:33–9.

[23] Irby C. Which Plastics Are FDA Compliant for Food Storage? (2020). Available online at: https://www.plascene.com/which-plastics-are-fda-compliant-for-food-storage (accessed December 2022).

[24] Bhasin V Kirandeep Kaur Dhaliwal S Gautam A. Assessment of the knowledge and attitude regarding plastic use and its health effects among nursing students of selected nursing colleges of Ambala, Haryana. Medico-legal Update. (2021) 21:2479. 10.37506/mlu.v21i1.2479

[25] Alharbi MH Mumena WA Hammouda SA. Use of plastics with hot food among saudi pregnant women is associated with increased concentrations of A1C, thyroid-stimulating hormone, and homocysteine and decreased concentrations of vitamins and minerals. Nutrients. (2020) 12:2609. 10.3390/nu12092609 32867150 PMC7551572

[26] Astolfi ML Castellani F Avino P Antonucci A Canepari S Protano C. Reusable water bottles: release of inorganic elements, phthalates, and bisphenol A in a “Real Use” simulation experiment. Separations. (2021) 8:1–13. 10.3390/separations8080126

[27] Mortula MM. Health risk assessment of PET bottles in GCC. Int Scholarly Sci Res Innov. (2013) 7:276–2.

[28] Barbir J Leal Filho W Salvia AL Fendt MTC Babaganov R Albertini MC. Assessing the levels of awareness among European citizens about the direct and indirect impacts of plastics on human health. Int J Environ Res Public Health. (2021) 18:3116. 10.3390/ijerph18063116 33803525 PMC8003071

[29] Du Preez M Van der Merwe D Wyma L Ellis SM. Assessing knowledge and use practices of plastic food packaging among young adults in South Africa: concerns about chemicals and health. Int J Environ Res Public Health. (2021) 18:10576. 10.3390/ijerph182010576 34682322 PMC8535462

[30] *Facebook users in Egypt in May* (2022). Available online at: https://napoleoncat.com/stats/facebook-users-in-egypt/2022/05/ (accessed December 2022).

[31] Filho WL Salvia AL Bonoli A Saari UA Voronova V Klõga M. An assessment of attitudes towards plastics and bioplastics in Europe. Sci Total Environ. (2021) 755:142732. 10.1016/j.scitotenv.2020.142732 33092843

